# Landscape structure affects the sunflower visiting frequency of insect pollinators

**DOI:** 10.1038/s41598-021-87650-9

**Published:** 2021-04-14

**Authors:** Károly Lajos, Ferenc Samu, Áron Domonkos Bihaly, Dávid Fülöp, Miklós Sárospataki

**Affiliations:** 1 Department of Zoology and Ecology, Hungarian University of Agriculture and Life Sciences, Páter Károly utca 1, Gödöllő, 2100 Hungary; 2 Centre for Agricultural Research, Plant Protection Institute, Eötvös Lóránd Research Network, Herman Ottó út 15, Budapest, 1022 Hungary

**Keywords:** Agroecology, Ecosystem services

## Abstract

Mass-flowering crop monocultures, like sunflower, cannot harbour a permanent pollinator community. Their pollination is best secured if both managed honey bees and wild pollinators are present in the agricultural landscape. Semi-natural habitats are known to be the main foraging and nesting areas of wild pollinators, thus benefiting their populations, whereas crops flowering simultaneously may competitively dilute pollinator densities. In our study we asked how landscape structure affects major pollinator groups’ visiting frequency on 36 focal sunflower fields, hypothesising that herbaceous semi-natural (hSNH) and sunflower patches in the landscape neighbourhood will have a scale-dependent effect. We found that an increasing area and/or dispersion of hSNH areas enhanced the visitation of all pollinator groups. These positive effects were scale-dependent and corresponded well with the foraging ranges of the observed bee pollinators. In contrast, an increasing edge density of neighbouring sunflower fields resulted in considerably lower visiting frequencies of wild bees. Our results clearly indicate that the pollination of sunflower is dependent on the composition and configuration of the agricultural landscape. We conclude that an optimization of the pollination can be achieved if sufficient amount of hSNH areas with good dispersion are provided and mass flowering crops do not over-dominate the agricultural landscape.

## Introduction

The successful cultivation of insect-pollinated mass-flowering crops (MFCs) largely depends on the visiting frequency of different insect pollinator species in the blooming period of these crops^1–3^. It has been shown that the structure of the landscape surrounding fields of MFCs can have a significant impact on pollinator flower-visiting frequency and thus the pollination success of these crops^2,3^. Examples for pollinator-dependent MFCs are oilseed rape (*Brassica napus* L.), sunflower (*Helianthus annuus* L.) or orange (*Citrus* × *sinensis* L.), which crops are cultivated in monoculture over large areas and bloom synchronously over a short period^3^. In the last few decades, there has been a decline in the abundance and species richness of wild insect pollinators globally^4^. One of the main causes of this process is the homogenisation of agriculturally used landscapes into monocultures with large field sizes at the expense of non-crop areas, resulting in the fragmentation and degradation of semi-natural habitats (SNHs)^4–8^. Examples for woody SNHs are hedgerows, lines of trees, wood- or shrubland areas, whereas herbaceous SNHs comprise habitats such as grass-strips, grassy field margins or grasslands^9^. SNHs in agricultural landscapes can be characterised by significantly lower mechanical and chemical disturbance, and therefore higher stability and plant diversity than crop areas. These areas serve as source habitats for wild pollinators. The decline in source habitat area and subsequent decrease of pollinator densities can lead to significant reductions in the yield of crops that require insect pollination^5,10–13^. Therefore, both for the prevention of further biodiversity loss and for securing pollination of our crops, it is our basic interest to understand the landscape scale dynamics of pollinators and to apply landscape management measures that help to sustain their populations. The influence of landscape structure is especially strong on wild pollinators, which—unlike managed honey bees—permanently live in the area surrounding the MFCs.

Most wild insect pollinator species are sensitive to the amount, composition and configuration of SNHs in the agricultural landscape, because they provide foraging and shelter areas as well as nesting resources for wild pollinator groups^2,3,14^. Especially herbaceous semi-natural habitat (hSNH) areas play a crucial role in wild pollinator distribution in the landscape. These areas can support wild pollinator communities throughout the year if the diversity of flowering plants provide nectar and pollen resources over the whole season^15–17^. There have been several studies, which used spatial analysis tools to investigate the effects of the composition and configuration of different landscape elements, primarily SNH patches. For example, habitat area, expressed as the proportion of SNH patches in the landscape, significantly increases the abundance of different solitary pollinator guilds^18,19^. The capacity of such SNH patches to be a source of pollinators is partly area-related, since habitat area sets the carrying capacity for wild pollinators^20^. However, the spillover of wild pollinators from a source patch happens across the edges; therefore edge characteristics are also crucial parameters of spillover^21,22^. If we consider a large patch of a source habitat, then those wild pollinators that stay in the interior of the patch, i.e. their foraging range does not reach the edge of the patch, will not contribute to the spillover process. Similarly, the spatial configuration of source patches is also important, because it affects the mobility and foraging range of different pollinators by determining the probability of a pollinator reaching other habitat patches^23^. Studies carried out in Hungary also showed that a high habitat heterogeneity of the agricultural landscape, such as the presence of herbaceous and woody habitats, and also the inclusion of organic fields among the conventionally managed ones, positively influences the abundance and species richness of wild insect pollinators^24–26^.

Landscape structure can also have a negative impact on the pollinator populations, even apart from the obvious negative effect of the increase in non-flowering crop areas at the expense of SNHs. MFCs, such as oilseed rape and sunflower, offer rich nectar and pollen resource to pollinators, but only temporarily^27^. During blooming period these MFCs redistribute pollinator populations via spillover process from other patches. The distribution of pollinators arises from a dynamic process where different patches attract various proportions of a finite pollinator population^28^. From the perspective of a given patch other patches may play a competing role by depleting the available pollinator population, but in other circumstances may concentrate pollinators from a wider area through resource concentration effect^29^. These processes make the role of MFCs ambiguous in enhancing pollinator populations, and likely dependent on the land-cover and configuration of MFCs and SNH patches in the given landscape.

Besides the composition and configuration of different landscape elements, the possible influence of different spatial scales also has to be taken into account. For example, the results of Tscheulin et al.^30^, who examined the impact of landscape structure on the abundance and species richness of different wild bee species in olive groves, showed that the spatial scales where the strongest correlations occurred corresponded with the size of the investigated wild bee species. The abundance and species richness of wild bees in the olive groves was positively influenced by both area and aggregation related metrics of SNH patches. A meta-analysis of Garibaldi et al.^10^ also demonstrated that the visitation rate of all pollinators, except for honey bees, decreased with distance from natural areas. In addition, a marked scale-dependency was found in the mobile group of bumble bees (*Bombus* sp.), where positive effects of SNHs occurred only at > 500 m spatial scales^26^. All these studies indicate that the size and scale-dependent configuration of SNH patches and also of other types of habitats have a strong influence on the abundance and species richness of wild insect pollinators. See Kennedy, et al.^31^ for a global quantitative synthesis using a complex configuration metric.

Among MFCs, sunflower (*Helianthus annuus* L.) is grown on more than 4 million hectares in the European Union (= EU-28), making it the second most important oil crop after rape and turnip rape^32^. In Hungary, sunflower is the most important oil crop, cultivated on more than 500.000 hectares since 2005^33^. Even though it is capable of self-pollination, cross-pollination by insect pollinators results in better quality seeds and higher yields^34–36^, up to 40% at field scale^37^. Although, globally viewed, managed honey bee colonies are the main insect pollinators of sunflower (e.g.^38–45^), wild bees, whose role has been less investigated, were found to mostly indirectly, but significantly affect sunflower yield, by increasing the pollination efficiency of honey bees^44,46,47^. Such an effect seems to be aggravated in hybrid sunflower systems where male-sterile (i.e. female only) plants require a polliniser movement for effective pollination^38,39,42,44^. Therefore, in sunflower production in general, it is important to know, which aspects of landscape structure enhance the abundance and species richness of wild insect pollinators. The influence of landscape characteristics on wild pollinators visiting sunflower fields, however, has been rarely investigated^24,48^. It has been shown that a higher amount of SNHs in the landscape surrounding sunflower fields strongly enhances the abundance and species richness of wild insect pollinators^49^. However, the blooming of MFCs can also lead to a depletion of wild pollinator density in the surrounding SNHs, due to their strongly attractive effect on the pollinators^3^, which effect has not been investigated specifically for sunflower fields. For this reason, it is important to fill in the knowledge gap, how the different composition and configuration of the agricultural landscape affect the pollination efficiency of this crop, with special regard that landscape effects might differ between pollinator groups with different foraging ranges and other functional traits.

We focused our study on the most important groups of pollinators of sunflower fields in Hungary: honey bees, wild bees and non-bee insect pollinators. Different pollinator groups interact with landscape structure differently pertaining to their functional traits, such as foraging range, nesting habitats, sociality, and, importantly, if their populations are managed by humans. Functionally, management is an important trait, because bee keepers control managed honey bee population sizes and also their landscape distribution by moving the hives, modifying this way their interaction with the landscape^10^. On the other hand, in wild insect pollinators, which includes both wild bees and non-bee pollinators, populations are governed by natural dynamics being dependent on the amount and distribution of resources provided in the given landscape.

Foraging behaviour covers a range of functional traits, in which respect the most important distinction between the studied groups is whether the species are central place foragers or vagrant, nomadic foragers. Honey bees and all wild bees considered here fall into the central place forager category, because they are all nesting species and all their foraging trips are centred around the nest, irrespective of whether they are social or solitary species. In contrast to bee pollinators, non-bee pollinators encountered in this study were non-nesting. These pollinators follow a nomadic lifestyle and move on freely from one habitat patch to the next one, meaning that even smaller foraging ranges can add up over time to a larger habitat area covered by their pollination action. In this regard foraging range is a more important trait for central place foragers, because the necessity to return to their nest after a foraging trip will limit their interaction with landscape structure to an area set by this range.

In this study we wanted to know how the structure of our studied landscape, that is the amount and spatial configuration of hSNH patches and sunflower fields occurring in the landscape, affect the sunflower visiting frequency of different pollinator groups. We distinguished three major pollinator groups: managed honey bees, wild bees and non-bees. We also wanted to test the scale-dependency of the landscape effects. For this, we recorded the flower visitation by pollinators in 36 sunflower fields and determined the spatial properties of the two studied landscape elements in increasing sectors around these fields. Our findings indicated that both area and/or dispersion of hSNH patches positively affected sunflower visiting frequency in all pollinator groups. However, for wild bees we also found considerably negative effects of the edge density of sunflower fields. Another interesting finding of our study was that honey bee visitation in sunflower fields was enhanced by an increasing spatial proportion and dispersion of hSNH patches, despite the fact that their hives were artificially maintained and positioned in the landscape.

## Results

### Pollinator assemblage and local effects

Over the two sampling years we observed 2993 potential pollinators on the investigated sunflower heads (Table S1 A). The vast majority (85.2%) of them were managed honey bees (*Apis mellifera* L.). In comparison, the number of wild pollinators was rather low, with wild bees only making up 7.8% (n = 233) and non-bee pollinators 7.0% (n = 209) of the total number of observed pollinators (for detailed numbers see Table S1 A). We were able to identify 103 wild bee specimens at species level. Most of the individuals belonged to the *Lasioglossum* genus (*L. lineare*, *L. malachurum* and *L. politum*), followed by *Bombus* spp. (*B. terrestris*, *B. lapidarius* and *B. pascuorum*), *Andrena flavipes* and *Halictus sexcinctus* (Table S1 B)*.* However, we could not collect and thus were not able to identify the rest of the observed wild bee pollinators (n = 130) at species level. Among non-bee pollinators (Table S1 B), we found flies (Diptera), mainly hoverflies (Syrphidae: Diptera), and also observed few butterflies (Lepidoptera). But the majority (n = 166) of non-bee pollinators, predominantly beetles (Coleoptera) and bugs (Heteroptera), was not further classified.

From the field variables only the intensity of cloud cover had a significant, negative effect on the sunflower visiting frequency of wild bees (z-value = − 3.131; *p*-value = 0.002; Table S2 A). The distance from field edge significantly affected honey bees as well as wild bees (Table S2 B), which showed a decreasing sunflower visiting frequency with increasing distance from field edge (z-value = − 2.468, *p*-value = 0.014 for honey bees; z-value = − 2.918, *p*-value = 0.004 for wild bees).

### Effects of hSNH patches and sunflower fields on the pollinator groups

The average proportion of hSNH patches for all 36 studied landscape sectors was nearly constant over all scales, ranging around 9–10%, while the proportion of sunflower fields dropped from nearly 60% at 150 m to ca. 25% at 750 m (Table S3).

**Figure 1:**
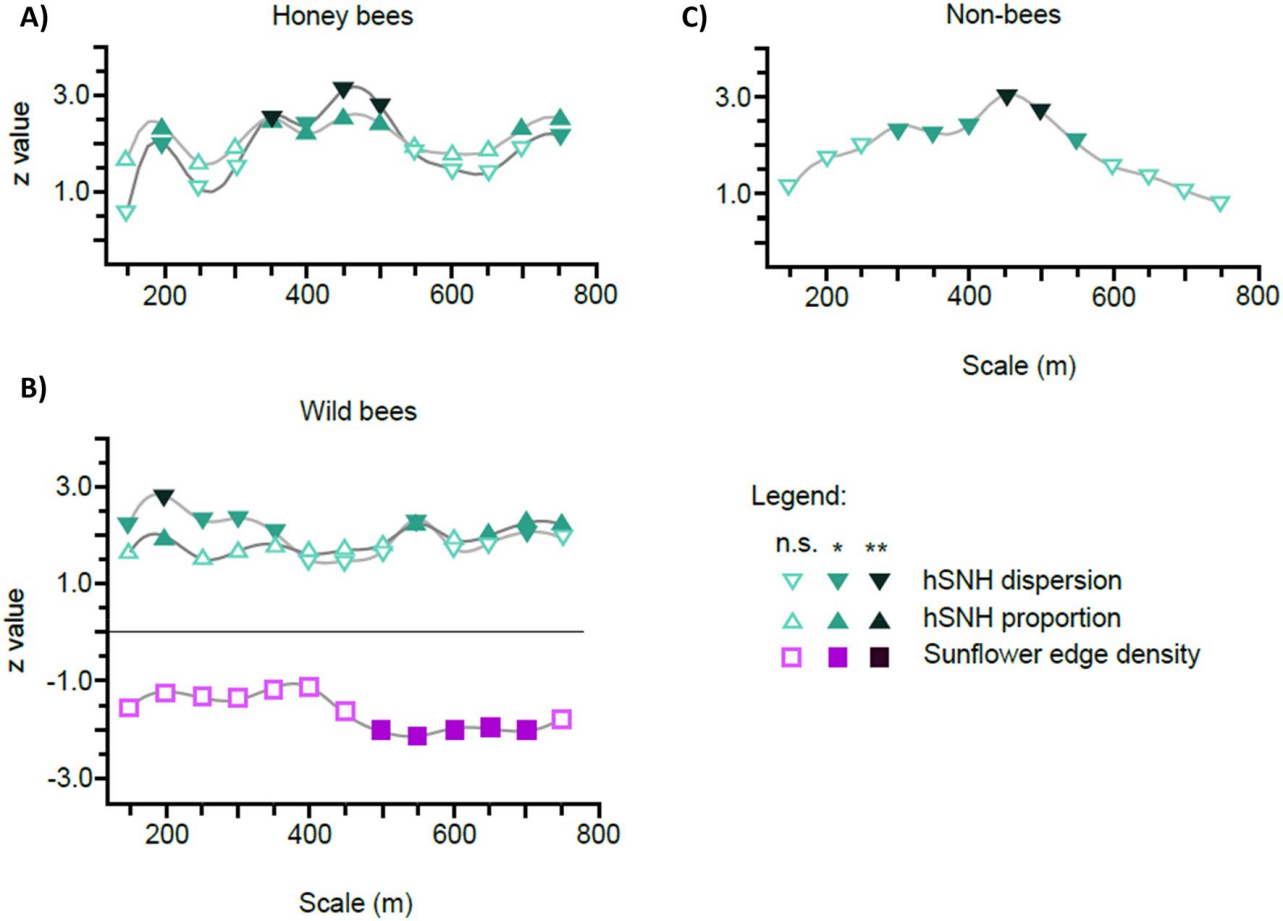
Z-values taken from generalized linear mixed models (GLMMs) assuming a Poisson distribution. The Poisson GLMMs tested for the effects of three different landscape metrics of hSNH patches and sunflower fields over 13 spatial scales (150–750 m) on the sunflower visiting frequency of (**A**) honey bees, (**B**) wild bees and (**C**) non-bees. From the three tested metrics only those having significant effects are presented in the figures. For the coefficients of the Poisson GLMMs with all metrics see Tables S4–S6. Trend lines were fitted among the points to make changes in the trend of the z-values more apparent. The intensity of coloration represents statistical significance (n.s. = not significant; * ≤ 0.05; ** ≤ 0.01).

Honey bees, the most abundant sunflower-visiting group, were significantly influenced by the proportion and dispersion of hSNH patches (Fig. 1A; Table S4 A). The spatial properties of sunflower fields in the landscape exerted no significant influence (Table S4 B). The positive effect of hSNH patches was scale-dependent and the strongest at scales between 350 and 500 m.

Considering the two groups of wild pollinators, that is wild bees and non-bee insect pollinators, the Poisson GLMMs revealed that the landscape elements differently affected their sunflower visiting frequency. Similarly to manged honey bees, wild bees were also positively affected by both the proportion and dispersion of hSNH patches. These effects were significant over two distinct ranges of spatial scales, with a gap between 400 and 550 m, where none of the two metrics had significant effects (Fig. 1B; Table S5 A). The impact of the dispersion of hSNH patches was more scale-dependent than of their proportion. While the effects of the dispersion of hSNH patches were significant at smaller scales (150–350 m) and at single larger scales (550 and 700 m), the impact of the proportion of hSNH patches was, except for a significant effect at 200 m, more significant at larger scales (550 and 650–750 m). In contrast to the positive effects of hSNH patches, the edge density of sunflower fields negatively affected the visitation frequency of wild bees, being significant at larger scales (500–700 m). The effects of the proportion and dispersion of sunflower fields was not significant (Table S5 B).

In comparison to the group of wild bees, non-bees were only significantly affected by the dispersion of hSNH patches. This positive effect of the dispersion of hSNH patches was clearly scale-dependent, reaching a peak value at 450 m (Fig. 1C; Table S6 A). Similarly to the group of honey bees, the spatial properties of sunflower fields had no significant impact on non-bees (Table S6 B).

### Comparison of pollinators’ scale-dependent responses to hSNH patches and sunflower fields

**Figure 2:**
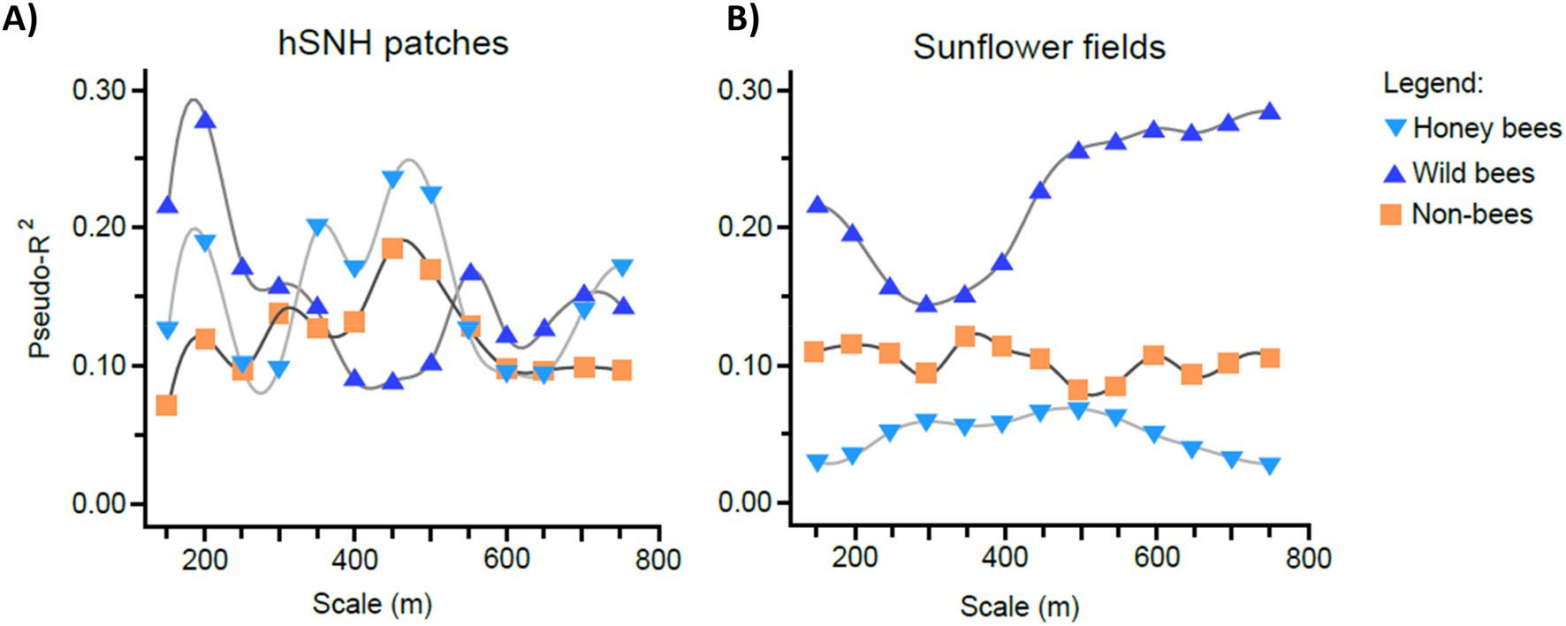
Explanatory power (= Pseudo-R^2^-values) of the Poisson GLMMs from Tables S4–S6, for (**A**) hSNH patches and (**B**) sunflower fields over 13 spatial scales (150–750 m). Trend lines were fitted among the points to make changes in the trend of the Pseudo-R^2^-values more apparent. The three studied pollinator groups are marked with different colours.

We compared the explanatory power of the GLMMs along the investigated scales, separately for hSNH patches and sunflower fields. These show a higher scale-dependency in hSNH influence than in sunflower field influence (Fig. 2A,B). The strongest effect of hSNH patches was found for wild bees, but only at close range (200 m; Pseudo-R^2^ = 0.281). The effects of hSNH patches on honey bees was more or less trimodal, with moderate effects at close range (200 m; Pseudo-R^2^ = 0.191) and at the largest scale of 750 m (Pseudo-R^2^ = 0.174). However, the strongest effects occurred at a medium range of 450 m (Pseudo-R^2^ = 0.242). Non-bees were also affected the strongest at a medium scale of 450 m (Pseudo-R^2^ = 0.191).

Sunflower fields had the strongest effects on the group of wild bees, especially at scales over 450 m up to the highest investigated scale of 750 m, with Pseudo-R^2^ values keeping a high level of 0.233–0.283 in that range. Compared to wild bees, the effects of sunflower fields were considerably weaker for both honey bees (highest Pseudo-R^2^ value of 0.068 at 500 m) and non-bees (highest Pseudo-R^2^ value of 0.123 at 350 m). These effects were also clearly less scale-dependent than for wild bees.

## Discussion

The hSNH patches in the studied landscape overall had positive effects on the sunflower visitation of all pollinator groups. The proportion and dispersion of hSNH patches increased sunflower visitation of honey bees and wild bees, whereas visitation frequency of non-bee pollinators was positively affected by the dispersion of hSNH patches alone. Intensive land use in the agricultural region of our study meant that there was a relatively low cover of hSNH patches (c. 10% at each scale) in the landscape sectors around the focal sunflower fields. This low proportion of hSNH patches, which serve as foraging, shelter and nesting areas for many wild bee species^2,3,14^, might also explain the low abundance of wild bees encountered at our study sites. Such a low number of wild bees coupled with a strong dominance of managed honey bees is typical to many agricultural landscapes^25,50,51^. Since we expected a spillover process from semi-natural habitats, the positive effects for the wild pollinator groups were anticipated. However, the positive effect of hSNH patches on honey bees came rather surprisingly, because we hypothesised that positioning of their hives artificially by humans would override landscape effects.

The scale-dependency of the positive effects of hSNH patches (Figs. 1, 2) more or less coincided with the foraging ranges of the observed bee pollinators (Table 1). Studies dealing with the question of foraging range of pollinators found a positive relationship between the body size of pollinators and their foraging distances^52,53^. Table 1 gives an overview of different foraging ranges found in bees in various case studies. Such congruency suggests that the spatial properties of hSNH patches in the landscape may benefit the various pollinator groups in a complementary way, with hSNH patches at different spatial scales having the greatest positive effects on specific groups of pollinators. Although not addressed directly in the present study, among the different bee groups the level of sociality may further influence these pollinators’ interaction with the landscape through social information exchange, such as in honey bees, or memorising revisited resource locations, which has been shown for bumble bees and other wild bees^54,55^.

**Table 1:** Literature references on mean and maximal foraging distances of some bee species, which were observed and identified in the focal sunflower fields during this study.

Bee species	Mean foraging distance [reference]	Maximal foraging distance [reference]
*Andrena flavipes*	150 m^52^	260 m^56^ 415 m^52^
*Apis mellifera*	800 m^57^ 1074–1408 m^58^ 5500 m^59^	1100 m^60^
*Bombus lapidarius*	< 500 m^61^	> 300 m^62^ 450 m^63^ 1500 m^61^
*Bombus pascuorum*		449 m^63^
*Bombus terrestris*	270 m^64^ 500–1000 m^61^	300–325 m^62^ 758 m^63^ 800 m^64^ 1500 m^65^ 1750 m^61^
*Lasioglossum malachurum*		500 m^53^

In the case of wild bees we expected the clearest manifestation of landscape effects, because here human management does not directly interfere with their distribution and central place foraging bounds the group more heavily to the given landscape. Mean foraging range of solitary wild bees with small to medium body size have been shown to fall in the 100–300 m range, but maximal distances covered during a foraging trip can be over 1 km^66^. Bumble bees (*Bombus* spp*.*) were the largest among wild bees in the present study. While bumble bees very effectively utilised resources within 500 m of colonies with a mean foraging distance of workers of only around 270 m^64^, this distance could extend to at least 1.5 km^65^. Sárospataki et al^26^ found that grassland patches positively affected species richness of bumble bees between 500 and 1000 m and their abundance at 2000 m. The majority of wild bees sampled in our study were small-bodied, projecting short or moderate foraging distances. Indeed, the strongest effects of the hSNH patches found in the present study were at the scales of 150–250 m, with a clear peak value at 200 m. However, an interaction between foraging movement and landscape structure means that pollinator distribution depends not only on foraging traits, but on the landscape characteristics, as well.

Pollinator foraging traits, such as the central place foraging mode of wild bees, might be in interaction with landscape composition and structure. Martin, et al.^23^ synthesising studies about 1515 landscapes across Europe, came to the conclusion that spillover of arthropods and arthropod driven services were the strongest when, similarly to our case, SNH landcover was low. Other, patch distribution related landscape characteristics, similarly to our findings, were also important factors for wild bees. In a study, conducted in landscapes in Southern Germany, patch density was positively associated with total wild bee richness^67^. In that study patch density was found to increase the amount of edges and corridors that could act as food and nesting resources and dispersal routes for wild bees. In a wider meta-analysis variation in interpatch distance was shown to be important determinant of social bee abundance^31^, indicating the overall importance of landscape configuration for wild bees.

Honey bees are the most abundant flower visitors worldwide^68^, and were by far the most prominent pollinators of sunflower in our study. With honey bees we can expect that apart from foraging traits and landscape characteristics, human management is also a decisive factor in their pollination efficiency. Honey bees, which can be regarded medium sized among bees, were found to have a mean foraging distance around 800 m, measured from their apiary of origin in a marking study by Hagler, et al.^57^. In the present study the strongest effects of hSNH patches on honey bees were between scales of 350 and 500 m, with a peak at 450 m, which is somewhat lower figure than foraging range reported in the literature (Table 1). However, for honey bees we did not expect any significant effects of hSNH patches, since honey bee workers spread out to flowering fields from artificially and temporarily placed hives. Our initial hypothesis was supported by a synthesis from 29 studies^10^. This review indicated that in contrast to various wild bee groups, whose abundance was influenced differently by isolation from florally diverse natural and semi-natural areas, honey bee visitation did not change with isolation. As opposed to these findings, our results indicated significant positive effects of hSNH patches, related to both their areal extent and level of dispersion in the landscape. We think that the dietary needs of honey bees can offer an explanation for the positive effects of hSNH patches. Nutritional requirements may influence how honey bees forage in landscapes with different floral resources, as collecting pollen from a wide diversity of plants improves their diet composition^69,70^. Several previous studies have already demonstrated that a monofloral diet can have negative impacts on honey bee immune health^71–75^. This improved nutrition secured by a bigger choice of pollen resources can lead to a larger number and also a higher survival rate of the offspring and result in larger hives^71,75^. Compensatory foraging by honey bees can secure essential aminoacid diversity, and likely other nutrients^70^. We suggest that the presence of hSNH patches which offer resources for compensatory feeding will also enhance flower visitation by honey bees in sunflower fields within their foraging range, especially if the florally diverse resources are limited in the landscape. Another possible explanation for this phenomenon could have been that beekeepers preferably put their hives in the vicinity of larger hSNH patches, leading to an accumulation of honeybees in landscape sectors, where the proportion of hSNH patches was large. This pattern, however, could not be verified as there were only two landscape sectors, where beehives were located within the boundaries of the mapped sectors, and the proportion of hSNH patches in these two sectors was actually well below average (c. 3% and 2% of the total landscape sector area at the largest scale of 750 m, respectively).

An interaction between honey bees and wild bees might be important for the success of pollination. Even though honey bees were by far the most abundant pollinators in our study, wild bees may still significantly affect sunflower yield. Pollinator species richness was shown to significantly increase sunflower seed set and production in different studies^46,47^. In a study in hybrid sunflower, where, similarly to our case the large majority of flower visits was by honey bees (72%), behavioural interactions between wild and honey bees increased pollination efficiency of honey bees up to five-fold^44^. In situations, when wild bees were rare, honey bee pollination on average produced three seeds per single visit. However, with higher wild bee abundance honey bee pollination efficiency increased strongly, up to 15 seeds per visit on average. From the study of^44^ it became apparent that the presence of wild bees disrupted the flower specialisation of honey bees. After interacting with a wild bee on a male flower 20% of honey bees moved to a female sunflower, whereas only 7% switched after interacting with another honey bee. Also in sunflower, the encounter of honey bees with other bee species, butterflies and moths significantly enhanced honey bee movement among sunflower heads^46^. Furthermore, honey bees after wild bee interaction carried significantly more pollen on their bodies^39^. These behavioural interactions effectively doubled honey bee pollination services on an average hybrid sunflower field^44^.

More sunflower fields in the landscape neighbourhood, expressed by the increasing edge density of the fields, significantly decreased wild bee visitation on the monitored sunflower heads in the focal fields. This effect was the strongest at larger spatial scales (500–700 m). The fact that edge density was the important variable, and not area per se of the surrounding sunflower fields, suggests that sunflowers closer to edges attracted wild bees more than those in the interior of fields. In other words, if sufficient resources were present close to field edges maybe it was not worthwhile to travel greater distances further into the fields for this central place foraging group. This reasoning was supported by the finding that an increasing distance of the monitored sunflower heads from the field edge resulted in significantly lower observed numbers of visiting wild bees, an effect that was also significant for honey bees. Similar observations have already been made in the study of Hevia et al.^34^, who also observed a significant decrease in the numbers of wild bees with increasing distance from the edge of sunflower fields. The visual counts of honey bees were, however, not affected by the distance from field edge in their case.

As opposed to edge density, an increasing areal proportion of MFCs was also reported to have negative effects on pollinators. Across six European regions densities of bumble bees, solitary bees, managed honey bees and hoverflies were negatively affected by the cover of MFCs in the landscape. In SNHs, densities of bumble bees declined with increasing cover of MFCs but densities of honey bees increased^3^. In a German study, at the landscape scale, flowering oilseed rape negatively affected bumble bee densities in SNHs, presumably due to dilution of pollinators, but had a positive effect after flowering, when bees moved back to these SNHs^2^. Despite the temporal increase in floral resources MFCs provide, they overall may limit the growth of pollinator populations, because they fail to provide resource continuity^15^ and suitable nest sites^3^. These results together support the ‘landscape-moderated concentration and dilution hypothesis’, which proposes that—such as we found, at least partially, in all pollinator groups—MFCs dilute the density of pollinators, thus weakening pollination services per unit area, but do not affect overall pollinator population size^28^.

### Conclusions

An increasing proportion and/or dispersion of hSNH patches had positive effects on all studied pollinator groups. All these effects were scale-dependent and corresponded well with the foraging ranges of the observed bee pollinators. Our analysis revealed that pollinator groups reacted to the presence of hSNH patches in a complementary way over the different spatial scales. This meant that these habitat areas had a beneficial effect at every spatial scale through enhancing one or the other pollinator group, which effect diminished only at distances further than 600 m from the focal fields. As opposed to hSNH patches, sunflower fields in the landscape exerted a negative effect on wild bees. Presumably a higher presence of sunflower diluted wild bee populations, which were bound to a certain area due to their short range central place foraging mode. On all other pollinator groups with larger foraging ranges the effect of sunflower fields was marginal. Our results clearly indicate that the pollination of sunflower is dependent on the composition and configuration of the agricultural landscape. An optimization of the pollination process can be achieved if a sufficient amount of hSNH areas with good dispersion is provided and sunflower fields do not over-dominate. Such landscape configurations promote the beneficial actions of various pollinator groups, which do not only react to landscape structure in a complementary way, but also positively interact with one another to increase pollination efficiency. This case study also points out that studying landscape diversity may uncover ways to landscape optimisation for the benefit of various ecosystem services and the preservation of biodiversity.

## Materials and methods

### Study area and sampling methods

The study area was located in the central part of Hungary (Jász-Nagykun-Szolnok county), in an intensively used agricultural landscape (Figure S1). The main crops in this region are winter wheat, sunflower and maize^33^. The field experiments were carried out in randomly selected sunflower fields over two years (2014 and 2015), with 18 fields examined in each year with permission of the land owners or farmers (Table S7 A). We made all observations and taking samples from the insect populations with maximal respect to animal welfare. All applicable international, national, and/or institutional guidelines for the care and use of animals were followed. Each of the 36 study fields was only sampled once. Field sizes ranged from 1.77 to 108.12 hectares. The fields were located at distances of at least 1.5 km to each other (twice the radius of the landscape sectors, see below). Pollinator data from half of these fields (n = 18 sunflower fields, year 2014) was reported in a Hungarian paper by Bihaly et al.^24^, where no scale-dependent landscape analysis was applied.

**Figure 3:**
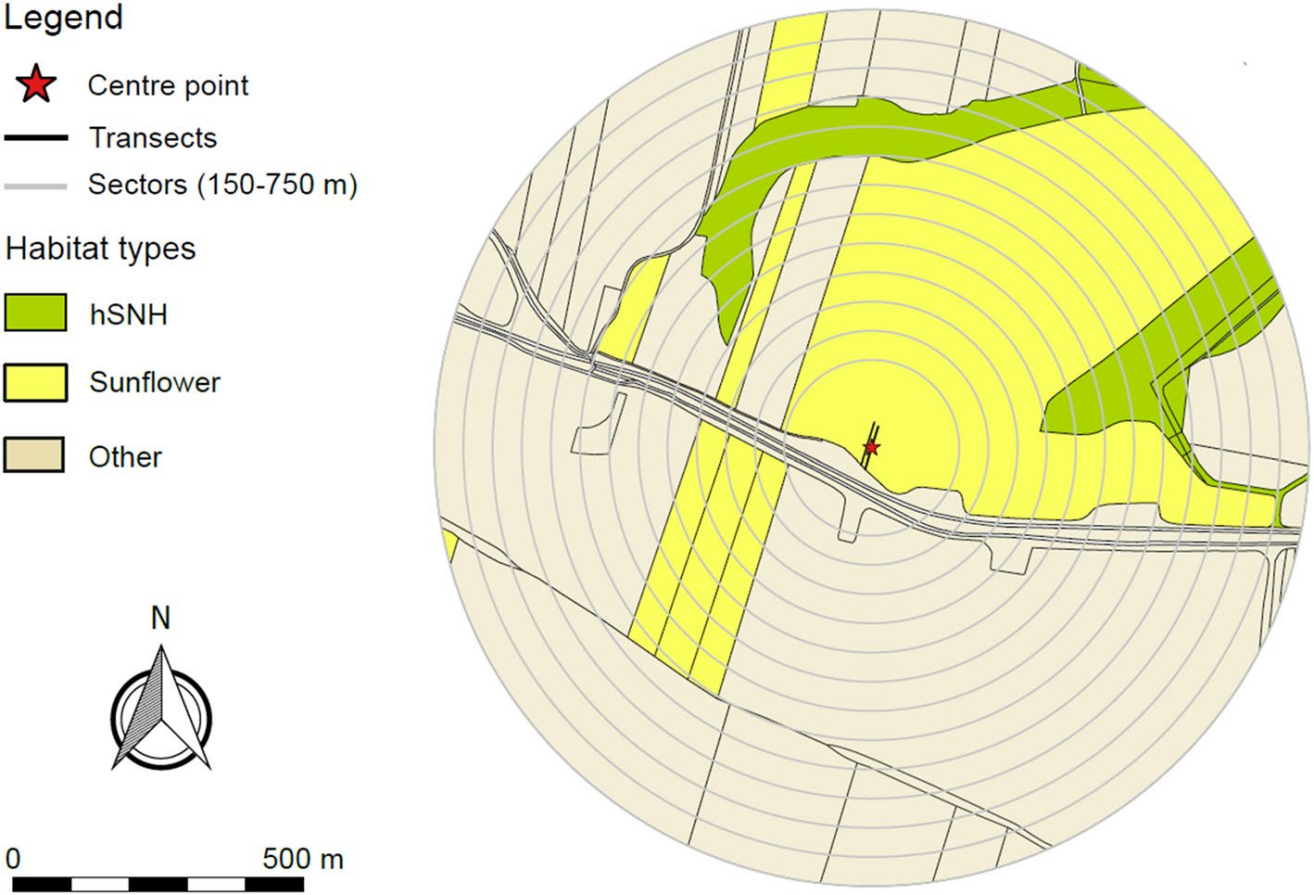
Example for a landscape sector with a composition of landscape elements typical for the study area. The vector map of the landscape sector was created with the software QGIS 2.18.9 (http://qgis.osgeo.org)^76^.

The sampling of the pollinators within the sunflower fields happened on July 14–17 in 2014 and on July 10–23 in 2015. On each occasion sampling was performed by visual observation between 9:00 and 17:00, adapted to the daily activity of bees. In each case the percentage of cloud cover and wind velocity was assessed. The sampling was carried out alongside two transects perpendicular to the field edge (Fig. 3), running parallel 10 m from each other. Along a single transect, there were four sampling points located at 5, 25, 50 and 75 m from the edge of the field. At each of these sampling points nine flowering sunflower heads were chosen for observation. These nine sunflower heads were monitored for 10 min by one person, and the pollinator insects, which landed on the flowers and thus may have been involved in the pollination process, were recorded by another person. The same sampling method was also used in Bihaly et al.^24^.

Since we considered the human management of honey bee colonies as a factor that may potentially override landscape effects, we made separate comparison between honey bees and all other non-managed wild pollinators. In the latter group, we introduced a second level of differentiation distinguishing between wild bees and non-bees. Honey bees and bumble bees were identified to species level at the sampling sites. Wild bee species, if we were able to collect them, were sent to a taxonomist for identification. If the sampling of the wild bee species did not happen immediately after their visitation of the sunflower heads, the time was stopped until the pollinator could be caught. The observation period was subsequently prolonged with the time, which was needed for the sampling. We tried to locate honey bee hives within the mapped landscape sector (up to 750 m). Hives occurred within this area in two sectors. In case of one of these sectors, honey bee hives were placed in the immediate neighbourhood of the sampled sunflower field. Since the number of honey bees sampled in this field was very large (n = 354), we excluded honey bee data for this field from further analyses.

### Mapping and calculation of landscape metrics

In order to reveal possible relationships between the spatial features of different habitat patches and these pollinator communities, the surroundings of the sampled sunflower fields (focal fields) were mapped in QGIS 2.18.9 (http://qgis.osgeo.org)^76^ using Google Satellite Images from 2014 and 2016 as base maps. The geo-referencing of the vector layers was done in the ETRS89/ETRS-LAEA (EPSG: 3035) coordinate reference system and the minimum area of all digitised patches was 100 m^2^. For testing possible scale-dependent effects, circles of 13 scales with differences of 50 m in radius were created around the centre point of one of the transects (at 37.5 m), ranging from 150 to 750 m (Fig. 3).

We distinguished two landscape element categories, as follows: (1) hSNH patches (herbaceous SNH patches), which were SNH patches with less than 30% shrub or tree canopy cover, like grass strips and pastures; (2) sunflower fields, fields with sunflower as crop in the given season, including the investigated focal fields. The proportion of the two landscape element types and their occurrence per landscape sector are listed in Table S3. The reason behind the separation of the sunflower fields from the other crops and not testing the effects of them was that we assumed that the spatial characteristics of the investigated, focal sunflower field and also that of other nearby sunflower fields may have significant effects on the abundance of the investigated pollinator groups, and they may also interact with one-another (e.g. compete for pollinators). Since none of the major crops besides sunflower was blooming at the time of the field experiments, we did not assume a similar possible influence of other crops. Sunflower fields around the focal fields were identified by ground observations and validated by using satellite images from Google Satellite, Landsat 8 or Sentinel 2A, recorded at multiple different dates between June and October for each sampling year. The correct identification of the hSNH patches was also double-checked using the open access database MePAR^77^.

As a next step, the vector layers of the surroundings were clipped by the circles with the largest radius (750 m). The resulting 36 landscape sectors were then rasterized with an output raster size of 1 × 1 m. The algorithm used was the GDAL command ‘Rasterize (vector to raster)’, executed as a batch process and with the output resolution set in map units per pixel. The resulting raster images were first sieved for small areas using the GDAL function ‘Sieve’ with a threshold set at 4 pixels, applying four connectedness (= 4-neighbourhood-rule), and then clipped by the circles with smaller radii, resulting in 36 raster images across 13 scales (from 150 to 750 m).

In order to quantify the spatial composition and configuration of the landscape sectors regarding the two landscape element types, we calculated three specific landscape metrics (also referred to as “metrics”) with FRAGSTATS v4.2.1^78^: (1) To quantify the composition of the landscape sectors we chose the metric ‘Percentage of Landscape’, which measures the proportional abundance of a particular patch type and thus quantifies the areal dominance of that patch type. (2) To quantify the configuration of the two landscape element types within the landscape sectors, we chose the metric ‘Edge Density’, which gives a measure of the edge length of a particular patch type related to the total landscape area. (3) Finally, we negated the values ‘Aggregation Index’, which quantifies both the spatial composition and configuration of a landscape unit, to create a metric, which we termed ‘Dispersion Index’. The “normal” ‘Aggregation Index’ quantifies the aggregation of the focal patch type using an adjacency matrix, giving the strength of the aggregation in percent. The value of this index is 0, when the focal patch type is maximally disaggregated and 100, when the patch type is maximally aggregated into a single, compact patch. In our case, the values of the ‘Dispersion Index’ are turned into the opposite and higher values mean that there is less aggregation or compactness of a particular patch type. So, basically, we created the new metric ‘Dispersion Index’ mirroring the “normal” ‘Aggregation Index’. We also simplified the names of two of these metrics, renaming ‘Percentage of Landscape’ into ‘proportion’ and the ‘Dispersion Index’ into ‘dispersion’. The definitions of these three metrics were taken from McGarigal^79^. All calculations were performed at the class level with an 8-cell neighbourhood rule. A good description about these metrics and the concept behind them can be found in the freely accessible lecture notes of Kevin McGarigal^80^.

### Data analysis and presentation

All analyses were carried out in R 3.6.3^81^. Spatial autocorrelation of the pollinator counts was tested by determining Moran’s I values using the R-package ‘ape’^82^ for each three pollinator groups. The coordinate reference system used for this calculation was ETRS89/ETRS-LAEA (EPSG:3035), which was also used for georeferencing the vector layers. We did not detect any spatial autocorrelation in the field counts of any pollinator group (Table S7 B).

To test the effects of field variables and landscape structure on the sunflower visiting frequency of the three pollinator groups, we applied generalised linear mixed models (GLMMs) assuming a Poisson distribution from the R package ‘lme4′^83^, with the site ID as a random factor in each case. The results of the Poisson GLMMs were plotted using the R package ‘ggplot2′^84^. All explanatory variables in the GLMMs were continuous ones, except for the study year, which was a categorical variable with the study year of 2014 as reference (Table S2 A). We used the sums of the field counts for the three pollinator groups in all these GLMMs, except for testing the effects of the distance from field edge, where we used the sums of the field counts for each four distances (Table S2 B). For analysing the effects of landscape structure on the sunflower visiting frequency of the studied pollinator groups, we added all three metrics mentioned above as explanatory variables in the Poisson GLMMs, for each two landscape element types separately. This separation was necessary because of the absence of hSNH patches in some landscape sectors at scales below 400 m, while sunflower fields were occurring in all of these sectors (Table S3).

The residuals of all Poisson GLMMs were checked for uniformity, dispersion and outliers using functions from the R package ‘DHARMa’^85^. In case the results of these tests would indicate a bad fit of a tested Poisson GLMM, we intended switch to a negative binomial GLMM. However, this change proved to be unnecessary, since the tests did not detect any significant deviations of the residuals for all tested Poisson GLMMs. All models were also tested for multicollinearity between the explanatory variables with variance inflation factors (VIFs) using the R package ‘car’^86^. The VIFs for the three tested metrics were below 2 at most spatial scales for hSNH patches, but over 2 at the majority of scales for sunflower fields in case of all three pollinator groups, indicating a high redundancy of the metrics for the latter landscape element.

## Supplementary information

Supplementary Information.

## Abbreviations

SNH: Semi-natural habitat
hSNH: Herbaceous SNH
MFC: Mass-flowering crop
