# Supplementary material for: Landscape structure affects the sunflower visiting frequency of insect pollinators
Source: Sci Rep. 2021 Apr 14;11:8147. doi: 10.1038/s41598-021-87650-9 (PMC8046751; doi:10.1038/s41598-021-87650-9)
Supplement: Supplementary file 1 — Supplementary Information. [file 41598_2021_87650_MOESM1_ESM.docx]

**Supplementary Information to: Landscape structure affects the sunflower visiting frequency of insect pollinators**

Károly Lajos^1^, Ferenc Samu^2^*, Áron Domonkos Bihaly^3^, Dávid Fülöp^4^, Miklós Sárospataki^5^

(1) PhD-student; Szent István University, Department of Zoology and Animal Ecology, Páter Károly utca 1; 2100 Gödöllő, Hungary;

(2) Scientific adviser; Centre for Agricultural Research, Plant Protection Institute, Herman Ottó út 15; 1022 Budapest, Hungary;

(3) PhD-student; Szent István University, Department of Zoology and Animal Ecology, Páter Károly utca 1; 2100 Gödöllő, Hungary;

(4) Research fellow; Centre for Agricultural Research, Plant Protection Institute, Herman Ottó út 15; 1022 Budapest, Hungary;

(5) Associate Professor; Szent István University, Department of Zoology and Animal Ecology, Páter Károly utca 1; 2100 Gödöllő, Hungary;

*Corresponding author: Samu.Ferenc@atk.hu


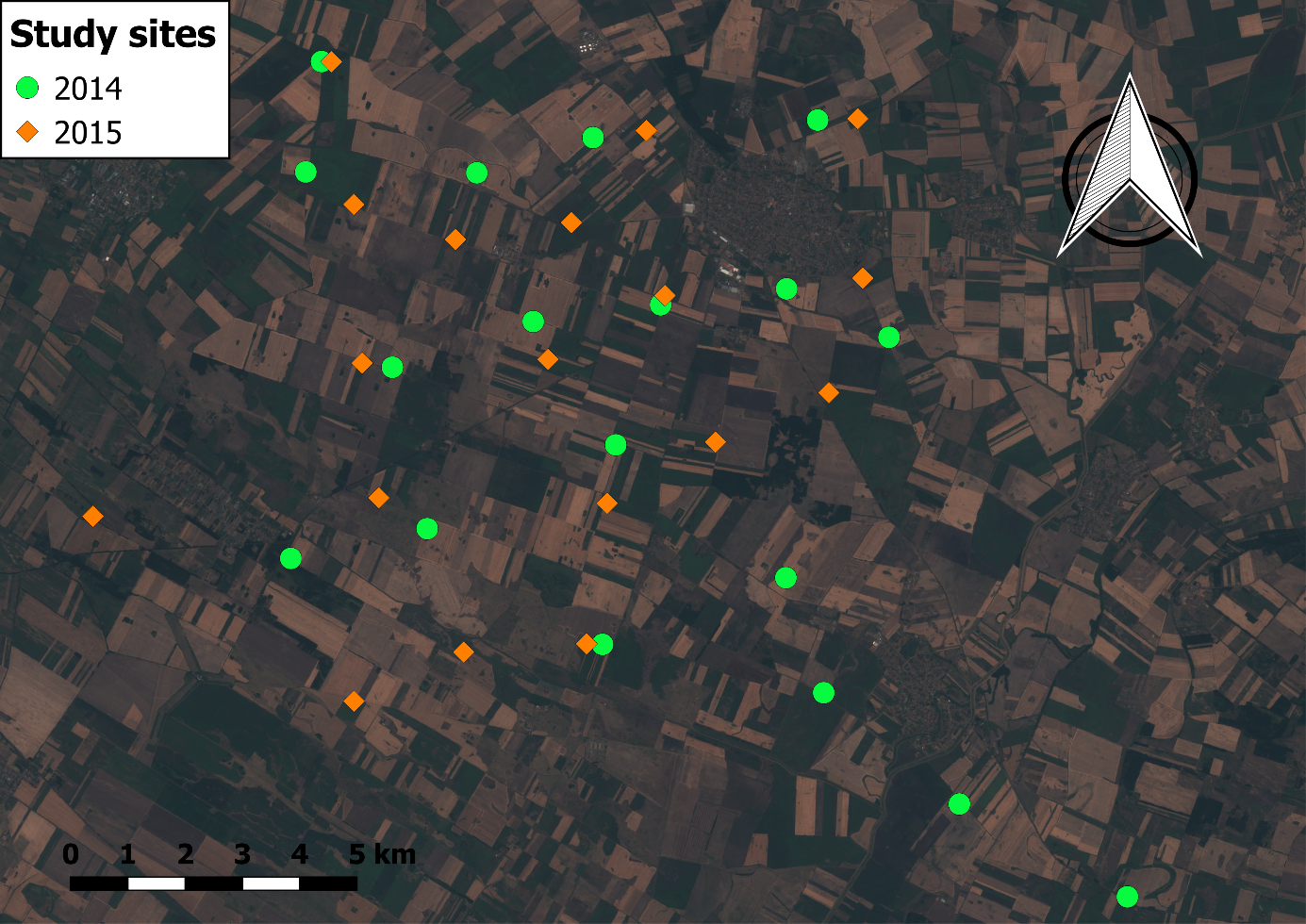


**Fig. S1.** Overview map of the study area with the positions of the sampled sunflower fields, marked with different colours and symbols for the two study years. The vector layer with the points of the study sites was created using the software QGIS 2.18.9 (<http://qgis.osgeo.org>) ^[69]^.The base map is an ESA Sentinel-2 Satellite Image from 2015-07-25.

**Table S1. A)** Total number and average (± SD) of field counts of insect pollinators visiting sunflower heads, grouped into three pollinator groups. The numbers are given for the two study years separately and also together. In case of the honey bees, the counts from one field were excluded due to the presence of a large apiary. The count data from 2014 were also used for analyses in Bihaly et al. (2018). **B)** Numbers of identified species (honey bees and wild-bees) and families (non-bees) for the three pollinator groups in **A)**. For further explanation, see text.

1. **Pollinator groups**

| Pollinator groups | 2014 | 2015 | Sum |
| --- | --- | --- | --- |
| **Honey bees – total number** | 1218 | 1333 | 2551 |
| **Honey bees – field average (± SD)** | 67.67 ± 35.11 | 78.41 ± 30.68 | 72.89 ± 33.00 |
| **Wild bees – total number** | 108 | 125 | 233 |
| **Wild bees – field average (± SD)** | 6.00 ± 5.78 | 6.94 ± 6.49 | 6.47 ± 6.08 |
| **Non-bees – total number** | 117 | 92 | 209 |
| **Non-bees – field average (± SD)** | 6.5 ± 3.38 | 5.11 ± 2.54 | 5.81 ± 3.03 |

1. **Identified pollinators**

| **Group** | **Taxonomic group** | **Number of observations** |
| --- | --- | --- |
| **Honey bees** | *Apis mellifera* | 2551 |
| **Wild bees** | *Andrena flavipes* | 9 |
|  | *Bombus terrestris* | 39 |
|  | *Bombus lapidarius* | 3 |
|  | *Bombus pascuorum* | 1 |
|  | *Halictus sexcinctus* | 1 |
|  | *Lasioglossum lineare* | 29 |
|  | *Lasioglossum malachurum* | 20 |
|  | *Lasioglossum politum* | 1 |
|  | Other wild bees | 130 |
| **Non-bees** | Syrphidae | 22 |
|  | Other Diptera | 19 |
|  | Lepidoptera | 2 |
|  | Other non-bees | 166 |

**Table S2.** Results of Poisson GLMMs testing for the effects of **A)** the study year, cloud cover and wind velocity and **B)** the distance from field edge on the sunflower visiting frequency of the three pollinator groups. The coefficients of the models’ intercepts are written in italics. Significant effects are marked bold.

1. **Effects of study year, cloud cover and wind velocity**

| **Pollinator group** | **Variable** | **Estimate** | **Std. Error** | **z-value** | **Pr(>\|z\|)** |
| --- | --- | --- | --- | --- | --- |
| **Honey bees** | *Intercept* | *4.165* | *0.197* | *21.184* | *<2e-16* |
|  | Year (ref.: 2014) | 0.115 | 0.183 | 0.627 | 0.530 |
|  | Cloud cover | -0.003 | 0.004 | -0.899 | 0.368 |
|  | Wind velocity | 0.025 | 0.078 | 0.325 | 0.745 |
| **Wild bees** | *Intercept* | *2.244* | *0.321* | *7.001* | *0.000* |
|  | Year (ref.: 2014) | -0.203 | 0.301 | -0.675 | 0.500 |
|  | Cloud cover | -0.021 | 0.007 | **-3.131** | **0.002** |
|  | Wind velocity | -0.073 | 0.128 | -0.568 | 0.570 |
| **Non-bees** | *Intercept* | *1.807* | *0.207* | *8.752* | *<2e-16* |
|  | Year (ref.: 2014) | -0.281 | 0.188 | -1.490 | 0.136 |
|  | Cloud cover | -0.001 | 0.004 | -0.134 | 0.894 |
|  | Wind velocity | 0.038 | 0.080 | 0.471 | 0.638 |

1. **Distance effects**

| **Pollinator group** | **Variable** | **Estimate** | **Std. Error** | **z-value** | **Pr(>\|z\|)** |
| --- | --- | --- | --- | --- | --- |
| **Honey bees** | *Intercept* | *2.869* | *0.089* | *32.336* | *<2e-16* |
|  | Distance | -0.002 | 0.001 | **-2.468** | **0.014** |
| **Wild bees** | *Intercept* | *0.566* | *0.178* | *3.175* | *0.002* |
|  | Distance | -0.007 | 0.002 | **-2.918** | **0.004** |
| **Non-bees** | *Intercept* | *0.266* | *0.138* | *1.935* | *0.053* |
|  | Distance | 0.002 | 0.003 | 0.585 | 0.559 |

**Table S3.** Proportions of the two different landscape element types calculated over 13 spatial scales (150-750 m). The number of landscape sectors, in which the studied landscape elements were occurring, is also listed.

| **Landscape element** | **Scale** | **Mean** | **Median** | **Minimum** | **Maximum** | **Occurrence** |
| --- | --- | --- | --- | --- | --- | --- |
| **hSNH patches** | 150 | 9.886 | 5.660 | 0.130 | 33.822 | 25 |
|  | 200 | 10.472 | 5.476 | 0.080 | 39.979 | 27 |
|  | 250 | 9.873 | 3.847 | 0.186 | 44.942 | 30 |
|  | 300 | 9.056 | 3.278 | 0.046 | 47.971 | 34 |
|  | 350 | 9.156 | 3.036 | 0.071 | 49.860 | 35 |
|  | 400 | 9.132 | 3.345 | 0.044 | 51.200 | 36 |
|  | 450 | 9.250 | 3.788 | 0.063 | 52.174 | 36 |
|  | 500 | 9.354 | 3.349 | 0.051 | 52.981 | 36 |
|  | 550 | 9.453 | 3.254 | 0.067 | 54.019 | 36 |
|  | 600 | 9.471 | 3.487 | 0.233 | 54.594 | 36 |
|  | 650 | 9.482 | 3.441 | 0.207 | 54.126 | 36 |
|  | 700 | 9.479 | 3.401 | 0.179 | 53.491 | 36 |
|  | 750 | 9.502 | 3.400 | 0.156 | 52.394 | 36 |
| **Sunflower fields** | 150 | 58.491 | 60.261 | 25.908 | 85.476 | 36 |
|  | 200 | 51.047 | 51.819 | 18.788 | 77.560 | 36 |
|  | 250 | 45.575 | 44.841 | 14.529 | 77.268 | 36 |
|  | 300 | 41.255 | 39.874 | 11.812 | 77.118 | 36 |
|  | 350 | 37.724 | 36.060 | 10.267 | 76.511 | 36 |
|  | 400 | 34.924 | 34.448 | 9.963 | 76.246 | 36 |
|  | 450 | 32.743 | 31.928 | 10.651 | 75.288 | 36 |
|  | 500 | 30.874 | 30.236 | 9.989 | 72.810 | 36 |
|  | 550 | 29.185 | 28.735 | 9.030 | 69.924 | 36 |
|  | 600 | 27.721 | 27.217 | 8.163 | 67.431 | 36 |
|  | 650 | 26.535 | 25.436 | 7.054 | 64.662 | 36 |
|  | 700 | 25.590 | 24.793 | 6.428 | 62.084 | 36 |
|  | 750 | 24.792 | 24.132 | 6.119 | 59.663 | 36 |

**Table S4.** Coefficients of Poisson GLMMs analysing the effects of the proportion, edge density and dispersion of **A)** hSNH patches and **B)** sunflower fields on the sunflower visiting frequency of honey bees over 13 spatial scales (150-750 m). The coefficients of the intercepts are written in italics.

1. **hSNH patches**

| **Metric** | **Scale** | **Estimate** | **Std. Error** | **z value** | **Pr(>\|z\|)** |
| --- | --- | --- | --- | --- | --- |
| ***Intercept*** | *150* | *4.214* | *0.093* | *45.375* | *0.000* |
|  | *200* | *4.214* | *0.084* | *50.232* | *0.000* |
|  | *250* | *4.273* | *0.082* | *51.940* | *0.000* |
|  | *300* | *4.205* | *0.080* | *52.547* | *0.000* |
|  | *350* | *4.188* | *0.073* | *57.243* | *0.000* |
|  | *400* | *4.155* | *0.078* | *53.121* | *0.000* |
|  | *450* | *4.160* | *0.075* | *55.678* | *0.000* |
|  | *500* | *4.160* | *0.076* | *54.963* | *0.000* |
|  | *550* | *4.182* | *0.080* | *52.150* | *0.000* |
|  | *600* | *4.193* | *0.082* | *50.849* | *0.000* |
|  | *650* | *4.191* | *0.084* | *50.014* | *0.000* |
|  | *700* | *4.230* | *0.088* | *47.935* | *0.000* |
|  | *750* | *4.266* | *0.095* | *44.758* | *0.000* |
| **Dispersion** | 150 | 0.013 | 0.026 | 0.510 | 0.610 |
|  | 200 | 0.051 | 0.025 | 2.016 | 0.044 |
|  | 250 | 0.027 | 0.025 | 1.098 | 0.272 |
|  | 300 | 0.034 | 0.024 | 1.431 | 0.152 |
|  | 350 | 0.061 | 0.024 | 2.568 | 0.010 |
|  | 400 | 0.061 | 0.026 | 2.369 | 0.018 |
|  | 450 | 0.073 | 0.024 | 3.070 | 0.002 |
|  | 500 | 0.071 | 0.025 | 2.872 | 0.004 |
|  | 550 | 0.056 | 0.031 | 1.823 | 0.068 |
|  | 600 | 0.051 | 0.034 | 1.502 | 0.133 |
|  | 650 | 0.049 | 0.035 | 1.377 | 0.169 |
|  | 700 | 0.093 | 0.049 | 1.909 | 0.056 |
|  | 750 | 0.122 | 0.056 | 2.176 | 0.030 |
| **Edge Density** | 150 | 0.000 | 0.001 | -0.181 | 0.856 |
|  | 200 | -0.001 | 0.001 | -0.545 | 0.585 |
|  | 250 | -0.002 | 0.002 | -1.152 | 0.249 |
|  | 300 | -0.001 | 0.002 | -0.705 | 0.481 |
|  | 350 | -0.002 | 0.002 | -0.949 | 0.343 |
|  | 400 | 0.000 | 0.002 | -0.222 | 0.825 |
|  | 450 | -0.001 | 0.002 | -0.356 | 0.722 |
|  | 500 | -0.001 | 0.002 | -0.423 | 0.672 |
|  | 550 | -0.001 | 0.002 | -0.645 | 0.519 |
|  | 600 | -0.002 | 0.003 | -0.785 | 0.433 |
|  | 650 | -0.003 | 0.003 | -1.061 | 0.289 |
|  | 700 | -0.004 | 0.003 | -1.506 | 0.132 |
|  | 750 | -0.006 | 0.003 | -1.874 | 0.061 |
| **Proportion** | 150 | 0.017 | 0.010 | 1.669 | 0.095 |
|  | 200 | 0.021 | 0.009 | 2.382 | 0.017 |
|  | 250 | 0.013 | 0.008 | 1.617 | 0.106 |
|  | 300 | 0.016 | 0.008 | 1.903 | 0.057 |
|  | 350 | 0.019 | 0.007 | 2.523 | 0.012 |
|  | 400 | 0.017 | 0.008 | 2.193 | 0.028 |
|  | 450 | 0.018 | 0.007 | 2.539 | 0.011 |
|  | 500 | 0.018 | 0.007 | 2.461 | 0.014 |
|  | 550 | 0.015 | 0.008 | 1.926 | 0.054 |
|  | 600 | 0.014 | 0.008 | 1.797 | 0.072 |
|  | 650 | 0.015 | 0.008 | 1.821 | 0.069 |
|  | 700 | 0.020 | 0.009 | 2.292 | 0.022 |
|  | 750 | 0.024 | 0.009 | 2.548 | 0.011 |

1. **Sunflower fields**

| **Metric** | **Scale** | **Estimate** | **Std. Error** | **z value** | **Pr(>\|z\|)** |
| --- | --- | --- | --- | --- | --- |
| ***Intercept*** | *150* | *4.258* | *0.362* | *11.760* | *0.000* |
|  | *200* | *4.294* | *0.200* | *21.459* | *0.000* |
|  | *250* | *4.191* | *0.123* | *34.013* | *0.000* |
|  | *300* | *4.202* | *0.095* | *44.185* | *0.000* |
|  | *350* | *4.198* | *0.083* | *50.853* | *0.000* |
|  | *400* | *4.176* | *0.082* | *50.768* | *0.000* |
|  | *450* | *4.148* | *0.086* | *48.458* | *0.000* |
|  | *500* | *4.125* | *0.091* | *45.547* | *0.000* |
|  | *550* | *4.103* | *0.099* | *41.297* | *0.000* |
|  | *600* | *4.093* | *0.113* | *36.116* | *0.000* |
|  | *650* | *4.094* | *0.127* | *32.190* | *0.000* |
|  | *700* | *4.104* | *0.131* | *31.338* | *0.000* |
|  | *750* | *4.096* | *0.139* | *29.567* | *0.000* |
| **Dispersion** | 150 | -0.290 | 1.759 | -0.165 | 0.869 |
|  | 200 | 0.124 | 1.429 | 0.087 | 0.931 |
|  | 250 | -0.812 | 1.141 | -0.712 | 0.477 |
|  | 300 | -0.518 | 1.014 | -0.511 | 0.610 |
|  | 350 | 0.017 | 0.856 | 0.019 | 0.985 |
|  | 400 | -0.008 | 0.816 | -0.010 | 0.992 |
|  | 450 | 0.479 | 0.795 | 0.602 | 0.547 |
|  | 500 | 0.845 | 0.938 | 0.901 | 0.368 |
|  | 550 | 0.980 | 1.167 | 0.839 | 0.401 |
|  | 600 | 0.637 | 1.298 | 0.491 | 0.623 |
|  | 650 | 0.301 | 1.341 | 0.224 | 0.823 |
|  | 700 | 0.074 | 1.205 | 0.061 | 0.951 |
|  | 750 | 0.139 | 1.226 | 0.114 | 0.910 |
| **Edge Density** | 150 | 0.001 | 0.007 | 0.161 | 0.872 |
|  | 200 | -0.002 | 0.007 | -0.219 | 0.826 |
|  | 250 | 0.004 | 0.007 | 0.583 | 0.560 |
|  | 300 | 0.003 | 0.007 | 0.455 | 0.649 |
|  | 350 | -0.001 | 0.007 | -0.106 | 0.915 |
|  | 400 | 0.000 | 0.007 | -0.046 | 0.963 |
|  | 450 | -0.005 | 0.008 | -0.581 | 0.561 |
|  | 500 | -0.008 | 0.009 | -0.832 | 0.405 |
|  | 550 | -0.010 | 0.011 | -0.862 | 0.388 |
|  | 600 | -0.008 | 0.013 | -0.618 | 0.536 |
|  | 650 | -0.005 | 0.013 | -0.374 | 0.709 |
|  | 700 | -0.002 | 0.013 | -0.147 | 0.883 |
|  | 750 | -0.002 | 0.013 | -0.150 | 0.881 |
| **Proportion** | 150 | -0.006 | 0.006 | -0.992 | 0.321 |
|  | 200 | -0.005 | 0.006 | -0.738 | 0.461 |
|  | 250 | -0.010 | 0.008 | -1.191 | 0.234 |
|  | 300 | -0.010 | 0.009 | -1.193 | 0.233 |
|  | 350 | -0.007 | 0.010 | -0.697 | 0.486 |
|  | 400 | -0.008 | 0.010 | -0.745 | 0.456 |
|  | 450 | -0.002 | 0.011 | -0.234 | 0.815 |
|  | 500 | 0.001 | 0.012 | 0.122 | 0.903 |
|  | 550 | 0.004 | 0.014 | 0.270 | 0.787 |
|  | 600 | 0.001 | 0.016 | 0.091 | 0.928 |
|  | 650 | -0.002 | 0.017 | -0.104 | 0.917 |
|  | 700 | -0.005 | 0.017 | -0.304 | 0.761 |
|  | 750 | -0.005 | 0.017 | -0.273 | 0.785 |

**Table S5.** Coefficients of Poisson GLMMs analysing the effects of the proportion, edge density and dispersion of **A)** hSNH patches and **B)** sunflower fields on the sunflower visiting frequency of wild bees over 13 spatial scales (150-750 m). The coefficients of the intercepts are written in italics.

1. **hSNH patches**

| **Metric** | **Scale** | **Estimate** | **Std. Error** | **z value** | **Pr(>\|z\|)** |
| --- | --- | --- | --- | --- | --- |
| ***Intercept*** | *150* | *1.476* | *0.293* | *5.042* | *0.000* |
|  | *200* | *1.489* | *0.178* | *8.350* | *0.000* |
|  | *250* | *1.559* | *0.219* | *7.124* | *0.000* |
|  | *300* | *1.607* | *0.148* | *10.853* | *0.000* |
|  | *350* | *1.576* | *0.152* | *10.370* | *0.000* |
|  | *400* | *1.590* | *0.151* | *10.510* | *0.000* |
|  | *450* | *1.599* | *0.150* | *10.635* | *0.000* |
|  | *500* | *1.603* | *0.149* | *10.736* | *0.000* |
|  | *550* | *1.636* | *0.143* | *11.443* | *0.000* |
|  | *600* | *1.657* | *0.149* | *11.117* | *0.000* |
|  | *650* | *1.674* | *0.150* | *11.174* | *0.000* |
|  | *700* | *1.740* | *0.157* | *11.053* | *0.000* |
|  | *750* | *1.779* | *0.172* | *10.339* | *0.000* |
| **Dispersion** | 150 | 0.130 | 0.057 | 2.282 | 0.023 |
|  | 200 | 0.151 | 0.054 | 2.805 | 0.005 |
|  | 250 | 0.115 | 0.050 | 2.310 | 0.021 |
|  | 300 | 0.102 | 0.043 | 2.353 | 0.019 |
|  | 350 | 0.099 | 0.048 | 2.065 | 0.039 |
|  | 400 | 0.072 | 0.049 | 1.488 | 0.137 |
|  | 450 | 0.068 | 0.046 | 1.471 | 0.141 |
|  | 500 | 0.079 | 0.047 | 1.668 | 0.095 |
|  | 550 | 0.121 | 0.052 | 2.336 | 0.020 |
|  | 600 | 0.102 | 0.059 | 1.727 | 0.084 |
|  | 650 | 0.113 | 0.061 | 1.843 | 0.065 |
|  | 700 | 0.179 | 0.087 | 2.059 | 0.039 |
|  | 750 | 0.198 | 0.102 | 1.932 | 0.053 |
| **Edge Density** | 150 | 0.000 | 0.002 | 0.180 | 0.857 |
|  | 200 | 0.002 | 0.003 | 0.585 | 0.558 |
|  | 250 | 0.000 | 0.003 | 0.045 | 0.964 |
|  | 300 | -0.001 | 0.003 | -0.157 | 0.875 |
|  | 350 | -0.001 | 0.004 | -0.324 | 0.746 |
|  | 400 | -0.001 | 0.004 | -0.297 | 0.767 |
|  | 450 | -0.001 | 0.004 | -0.373 | 0.709 |
|  | 500 | 0.000 | 0.004 | -0.047 | 0.963 |
|  | 550 | 0.000 | 0.004 | -0.001 | 0.999 |
|  | 600 | 0.000 | 0.005 | -0.055 | 0.956 |
|  | 650 | 0.000 | 0.005 | -0.033 | 0.974 |
|  | 700 | -0.002 | 0.005 | -0.392 | 0.695 |
|  | 750 | -0.004 | 0.006 | -0.645 | 0.519 |
| **Proportion** | 150 | 0.038 | 0.023 | 1.655 | 0.098 |
|  | 200 | 0.037 | 0.019 | 1.966 | 0.049 |
|  | 250 | 0.025 | 0.017 | 1.515 | 0.130 |
|  | 300 | 0.025 | 0.015 | 1.676 | 0.094 |
|  | 350 | 0.027 | 0.015 | 1.817 | 0.069 |
|  | 400 | 0.024 | 0.015 | 1.622 | 0.105 |
|  | 450 | 0.024 | 0.014 | 1.692 | 0.091 |
|  | 500 | 0.025 | 0.014 | 1.810 | 0.070 |
|  | 550 | 0.030 | 0.013 | 2.235 | 0.025 |
|  | 600 | 0.027 | 0.014 | 1.914 | 0.056 |
|  | 650 | 0.028 | 0.014 | 2.001 | 0.045 |
|  | 700 | 0.036 | 0.016 | 2.251 | 0.024 |
|  | 750 | 0.038 | 0.017 | 2.207 | 0.027 |

1. **Sunflower fields**

| **Metric** | **Scale** | **Estimate** | **Std. Error** | **z value** | **Pr(>\|z\|)** |
| --- | --- | --- | --- | --- | --- |
| ***Intercept*** | *150* | *2.564* | *0.629* | *4.080* | *0.000* |
|  | *200* | *1.821* | *0.331* | *5.509* | *0.000* |
|  | *250* | *1.731* | *0.202* | *8.571* | *0.000* |
|  | *300* | *1.688* | *0.160* | *10.569* | *0.000* |
|  | *350* | *1.635* | *0.141* | *11.601* | *0.000* |
|  | *400* | *1.596* | *0.142* | *11.274* | *0.000* |
|  | *450* | *1.534* | *0.148* | *10.384* | *0.000* |
|  | *500* | *1.466* | *0.156* | *9.385* | *0.000* |
|  | *550* | *1.409* | *0.168* | *8.376* | *0.000* |
|  | *600* | *1.359* | *0.187* | *7.289* | *0.000* |
|  | *650* | *1.305* | *0.206* | *6.334* | *0.000* |
|  | *700* | *1.298* | *0.211* | *6.157* | *0.000* |
|  | *750* | *1.301* | *0.221* | *5.877* | *0.000* |
| **Dispersion** | 150 | 2.031 | 3.166 | 0.642 | 0.521 |
|  | 200 | 0.624 | 2.523 | 0.248 | 0.804 |
|  | 250 | 0.735 | 1.964 | 0.374 | 0.708 |
|  | 300 | 0.801 | 1.763 | 0.455 | 0.649 |
|  | 350 | 0.016 | 1.511 | 0.010 | 0.992 |
|  | 400 | -0.187 | 1.422 | -0.131 | 0.896 |
|  | 450 | 0.355 | 1.344 | 0.264 | 0.792 |
|  | 500 | 1.087 | 1.551 | 0.701 | 0.483 |
|  | 550 | 1.781 | 1.873 | 0.951 | 0.342 |
|  | 600 | 1.583 | 2.012 | 0.787 | 0.431 |
|  | 650 | 1.661 | 2.043 | 0.813 | 0.416 |
|  | 700 | 1.301 | 1.821 | 0.714 | 0.475 |
|  | 750 | 0.579 | 1.851 | 0.313 | 0.754 |
| **Edge Density** | 150 | -0.019 | 0.013 | -1.536 | 0.125 |
|  | 200 | -0.015 | 0.012 | -1.212 | 0.226 |
|  | 250 | -0.016 | 0.012 | -1.338 | 0.181 |
|  | 300 | -0.017 | 0.012 | -1.376 | 0.169 |
|  | 350 | -0.014 | 0.012 | -1.122 | 0.262 |
|  | 400 | -0.014 | 0.013 | -1.121 | 0.262 |
|  | 450 | -0.023 | 0.014 | -1.679 | 0.093 |
|  | 500 | -0.031 | 0.015 | -2.032 | 0.042 |
|  | 550 | -0.039 | 0.018 | -2.122 | 0.034 |
|  | 600 | -0.039 | 0.020 | -1.960 | 0.050 |
|  | 650 | -0.041 | 0.021 | -1.974 | 0.048 |
|  | 700 | -0.039 | 0.020 | -2.015 | 0.044 |
|  | 750 | -0.034 | 0.020 | -1.734 | 0.083 |
| **Proportion** | 150 | 0.004 | 0.010 | 0.406 | 0.685 |
|  | 200 | 0.008 | 0.011 | 0.756 | 0.449 |
|  | 250 | 0.011 | 0.014 | 0.779 | 0.436 |
|  | 300 | 0.011 | 0.015 | 0.681 | 0.496 |
|  | 350 | 0.005 | 0.017 | 0.300 | 0.764 |
|  | 400 | 0.003 | 0.018 | 0.176 | 0.860 |
|  | 450 | 0.011 | 0.018 | 0.603 | 0.547 |
|  | 500 | 0.020 | 0.020 | 1.039 | 0.299 |
|  | 550 | 0.029 | 0.023 | 1.269 | 0.205 |
|  | 600 | 0.028 | 0.024 | 1.131 | 0.258 |
|  | 650 | 0.030 | 0.026 | 1.146 | 0.252 |
|  | 700 | 0.028 | 0.026 | 1.110 | 0.267 |
|  | 750 | 0.021 | 0.026 | 0.803 | 0.422 |

**Table S6.** Coefficients of Poisson GLMMs analysing the effects of the proportion, edge density and dispersion of **A)** hSNH patches and **B)** sunflower fields on the sunflower visiting frequency of non-bees over 13 spatial scales (150-750 m). The coefficients of the intercepts are written in italics.

1. **hSNH patches**

| **Metric** | **Scale** | **Estimate** | **Std. Error** | **z value** | **Pr(>\|z\|)** |
| --- | --- | --- | --- | --- | --- |
| ***Intercept*** | *150* | *1.790* | *0.111* | *16.063* | *0.000* |
|  | *200* | *1.771* | *0.101* | *17.541* | *0.000* |
|  | *250* | *1.729* | *0.095* | *18.120* | *0.000* |
|  | *300* | *1.702* | *0.087* | *19.602* | *0.000* |
|  | *350* | *1.723* | *0.084* | *20.597* | *0.000* |
|  | *400* | *1.692* | *0.086* | *19.693* | *0.000* |
|  | *450* | *1.701* | *0.083* | *20.479* | *0.000* |
|  | *500* | *1.703* | *0.084* | *20.283* | *0.000* |
|  | *550* | *1.723* | *0.085* | *20.167* | *0.000* |
|  | *600* | *1.732* | *0.088* | *19.720* | *0.000* |
|  | *650* | *1.728* | *0.090* | *19.282* | *0.000* |
|  | *700* | *1.738* | *0.096* | *18.097* | *0.000* |
|  | *750* | *1.735* | *0.105* | *16.530* | *0.000* |
| **Dispersion** | 150 | 0.032 | 0.030 | 1.065 | 0.287 |
|  | 200 | 0.048 | 0.028 | 1.703 | 0.089 |
|  | 250 | 0.050 | 0.026 | 1.915 | 0.055 |
|  | 300 | 0.055 | 0.023 | 2.358 | 0.018 |
|  | 350 | 0.054 | 0.024 | 2.225 | 0.026 |
|  | 400 | 0.059 | 0.025 | 2.364 | 0.018 |
|  | 450 | 0.068 | 0.023 | 3.002 | 0.003 |
|  | 500 | 0.063 | 0.023 | 2.697 | 0.007 |
|  | 550 | 0.058 | 0.028 | 2.062 | 0.039 |
|  | 600 | 0.049 | 0.032 | 1.559 | 0.119 |
|  | 650 | 0.045 | 0.033 | 1.350 | 0.177 |
|  | 700 | 0.054 | 0.050 | 1.081 | 0.280 |
|  | 750 | 0.048 | 0.059 | 0.809 | 0.418 |
| **Edge Density** | 150 | -0.001 | 0.001 | -1.110 | 0.267 |
|  | 200 | -0.002 | 0.002 | -1.219 | 0.223 |
|  | 250 | -0.001 | 0.002 | -0.417 | 0.677 |
|  | 300 | 0.001 | 0.002 | 0.518 | 0.605 |
|  | 350 | 0.000 | 0.002 | 0.232 | 0.817 |
|  | 400 | 0.001 | 0.002 | 0.483 | 0.629 |
|  | 450 | 0.000 | 0.002 | 0.207 | 0.836 |
|  | 500 | 0.000 | 0.002 | 0.166 | 0.868 |
|  | 550 | 0.000 | 0.002 | -0.040 | 0.968 |
|  | 600 | -0.001 | 0.003 | -0.288 | 0.773 |
|  | 650 | -0.002 | 0.003 | -0.568 | 0.570 |
|  | 700 | -0.003 | 0.003 | -0.879 | 0.379 |
|  | 750 | -0.003 | 0.003 | -0.948 | 0.343 |
| **Proportion** | 150 | 0.013 | 0.012 | 1.060 | 0.289 |
|  | 200 | 0.012 | 0.010 | 1.179 | 0.238 |
|  | 250 | 0.009 | 0.009 | 1.002 | 0.316 |
|  | 300 | 0.006 | 0.009 | 0.644 | 0.520 |
|  | 350 | 0.003 | 0.008 | 0.365 | 0.715 |
|  | 400 | 0.003 | 0.008 | 0.342 | 0.732 |
|  | 450 | 0.004 | 0.008 | 0.503 | 0.615 |
|  | 500 | 0.002 | 0.008 | 0.305 | 0.761 |
|  | 550 | 0.000 | 0.008 | 0.000 | 1.000 |
|  | 600 | -0.002 | 0.009 | -0.246 | 0.805 |
|  | 650 | -0.003 | 0.009 | -0.320 | 0.749 |
|  | 700 | -0.002 | 0.010 | -0.194 | 0.846 |
|  | 750 | -0.003 | 0.011 | -0.313 | 0.754 |

1. **Sunflower fields**

| **Metric** | **Scale** | **Estimate** | **Std. Error** | **z value** | **Pr(>\|z\|)** |
| --- | --- | --- | --- | --- | --- |
| ***Intercept*** | *150* | *1.346* | *0.413* | *3.259* | *0.001* |
|  | *200* | *1.515* | *0.215* | *7.059* | *0.000* |
|  | *250* | *1.571* | *0.144* | *10.886* | *0.000* |
|  | *300* | *1.637* | *0.109* | *14.972* | *0.000* |
|  | *350* | *1.687* | *0.090* | *18.803* | *0.000* |
|  | *400* | *1.718* | *0.087* | *19.831* | *0.000* |
|  | *450* | *1.734* | *0.090* | *19.349* | *0.000* |
|  | *500* | *1.743* | *0.095* | *18.295* | *0.000* |
|  | *550* | *1.773* | *0.102* | *17.347* | *0.000* |
|  | *600* | *1.825* | *0.114* | *16.021* | *0.000* |
|  | *650* | *1.848* | *0.128* | *14.466* | *0.000* |
|  | *700* | *1.870* | *0.132* | *14.176* | *0.000* |
|  | *750* | *1.890* | *0.141* | *13.414* | *0.000* |
| **Dispersion** | 150 | -1.419 | 1.792 | -0.792 | 0.428 |
|  | 200 | -1.027 | 1.425 | -0.721 | 0.471 |
|  | 250 | -1.242 | 1.288 | -0.964 | 0.335 |
|  | 300 | -1.148 | 1.176 | -0.977 | 0.329 |
|  | 350 | -1.196 | 0.984 | -1.215 | 0.224 |
|  | 400 | -1.030 | 0.920 | -1.120 | 0.263 |
|  | 450 | -0.811 | 0.896 | -0.905 | 0.365 |
|  | 500 | -0.764 | 1.044 | -0.732 | 0.464 |
|  | 550 | -1.256 | 1.251 | -1.004 | 0.315 |
|  | 600 | -1.923 | 1.391 | -1.383 | 0.167 |
|  | 650 | -1.840 | 1.412 | -1.304 | 0.192 |
|  | 700 | -1.868 | 1.290 | -1.449 | 0.147 |
|  | 750 | -2.055 | 1.338 | -1.536 | 0.124 |
| **Edge Density** | 150 | 0.002 | 0.007 | 0.277 | 0.782 |
|  | 200 | 0.001 | 0.007 | 0.145 | 0.885 |
|  | 250 | 0.003 | 0.007 | 0.445 | 0.656 |
|  | 300 | 0.003 | 0.007 | 0.460 | 0.646 |
|  | 350 | 0.004 | 0.007 | 0.478 | 0.633 |
|  | 400 | 0.002 | 0.008 | 0.318 | 0.750 |
|  | 450 | 0.000 | 0.008 | 0.059 | 0.953 |
|  | 500 | 0.000 | 0.010 | 0.040 | 0.968 |
|  | 550 | 0.005 | 0.012 | 0.411 | 0.681 |
|  | 600 | 0.011 | 0.013 | 0.868 | 0.385 |
|  | 650 | 0.012 | 0.014 | 0.845 | 0.398 |
|  | 700 | 0.013 | 0.013 | 0.965 | 0.334 |
|  | 750 | 0.015 | 0.013 | 1.093 | 0.274 |
| **Proportion** | 150 | 0.009 | 0.006 | 1.544 | 0.123 |
|  | 200 | 0.009 | 0.006 | 1.396 | 0.163 |
|  | 250 | 0.005 | 0.008 | 0.591 | 0.554 |
|  | 300 | 0.003 | 0.009 | 0.319 | 0.750 |
|  | 350 | 0.001 | 0.010 | 0.084 | 0.933 |
|  | 400 | 0.001 | 0.010 | 0.144 | 0.885 |
|  | 450 | 0.003 | 0.011 | 0.326 | 0.744 |
|  | 500 | 0.004 | 0.012 | 0.335 | 0.738 |
|  | 550 | 0.000 | 0.014 | -0.017 | 0.986 |
|  | 600 | -0.008 | 0.016 | -0.483 | 0.629 |
|  | 650 | -0.008 | 0.017 | -0.485 | 0.628 |
|  | 700 | -0.010 | 0.017 | -0.573 | 0.567 |
|  | 750 | -0.013 | 0.018 | -0.733 | 0.463 |

**Table S7.** **A)** Coordinates of the 36 study sites in Latitude/Longitude-format, presented as decimals in the WGS84 (EPSG:4326) coordinate reference system. **B)** Moran’s I, calculated for the three pollinator groups, in order to test for spatial autocorrelation between their field counts. The coordinate reference system used for this calculation was ETRS89/ETRS-LAEA (EPSG:3035). In case of the honey bees, the counts from one field were excluded due to the presence of a large apiary.

**A)** **Coordinates of the 36 study sites**

| **Site ID** | **Latitude** | **Longitude** |
| --- | --- | --- |
| A17_1 | 47.6060456 | 19.965311 |
| A1P | 47.6299893 | 19.9818172 |
| A2_1 | 47.6316643 | 19.9994852 |
| A2_3 | 47.6224115 | 20.0055563 |
| A4JD | 47.5848258 | 19.9816327 |
| A7 | 47.6137548 | 19.9916161 |
| B17_x1 | 47.5973266 | 19.8872062 |
| B2x | 47.6289289 | 19.9535961 |
| B4b | 47.6055938 | 19.9421824 |
| B5_1 | 47.5964778 | 19.9401836 |
| B6b | 47.5744073 | 19.9391166 |
| B6x | 47.5744932 | 19.9354145 |
| B7_1b | 47.6274946 | 19.9525911 |
| B8JD | 47.5668494 | 19.9904127 |
| C10JD | 47.5494286 | 20.0218883 |
| C11_1b | 47.6177276 | 19.8903712 |
| C14 | 47.6183834 | 19.8833806 |
| C5_1b | 47.6482473 | 19.8702699 |
| C9_3 | 47.6189752 | 19.9264869 |
| C9b | 47.6248768 | 19.9230395 |
| D6 | 47.6563468 | 19.9890162 |
| D8_2 | 47.6565472 | 19.9982987 |
| E11_1 | 47.6481043 | 19.9099543 |
| E13_2_2 | 47.6403224 | 19.9318846 |
| E16b | 47.6536091 | 19.9369214 |
| E17_1 | 47.6547283 | 19.9492608 |
| E3_1 | 47.6376987 | 19.9050267 |
| E6_2 | 47.6431549 | 19.8814628 |
| E8_1 | 47.6654894 | 19.873927 |
| E9_2 | 47.6654656 | 19.8762188 |
| I_7x | 47.5655016 | 19.8814629 |
| II_1 | 47.587851 | 19.8668074 |
| II_1_5 | 47.5731998 | 19.9069329 |
| II_3_1 | 47.592496 | 19.8984517 |
| K1 | 47.5944154 | 19.8209241 |
| MK4JD | 47.5349119 | 20.0608604 |

**B)** **Spatial autocorrelation (Moran’s I)**

| **Pollinator group** | **observed** | **expected** | **sd** | **p.value** |
| --- | --- | --- | --- | --- |
| **Honey bees** | -0.047 | -0.029 | 0.040 | 0.656 |
| **Wild bees** | -0.021 | -0.029 | 0.039 | 0.841 |
| **Non-bees** | -0.025 | -0.029 | 0.041 | 0.937 |
